# The Severity of Fatty Liver Disease Relating to Metabolic Abnormalities Independently Predicts Coronary Calcification

**DOI:** 10.1155/2011/586785

**Published:** 2011-12-27

**Authors:** Ying-Hsiang Lee, Yih-Jer Wu, Chuan-Chuan Liu, Charles Jia-Yin Hou, Hung-I. Yeh, Cheng-Ho Tsai, Shou-Chuan Shih, Chung-Lieh Hung

**Affiliations:** ^1^Cardiovascular Medicine, Department of Internal Medicine, Mackay Memorial Hospital, Taipei 10449, Taiwan; ^2^Department of Medicine, Mackay Medical College, Taipei, Taiwan; ^3^Department of Medicine, Mackay Medicine, Nursing and Management College, Taipei, Taiwan; ^4^Institute of Traditional Medicine, National Yang-Ming University, Taipei, Taiwan; ^5^Health Evaluation Center, Mackay Memorial Hospital, Taipei 10449, Taiwan; ^6^The Institute of Health Policy and Management, College of Public Health, National Taiwan University, Taipei, Taiwan; ^7^Department of Medical Technology, Yuanpei University of Science and Technology, Hsin-Chu, Taiwan; ^8^Department of Health Industry Management, Kainan University, Taoyuan, Taiwan

## Abstract

*Background*. Nonalcoholic fatty liver disease (NAFLD) is one of the metabolic disorders presented in liver. The relationship between severity of NAFLD and coronary atherosclerotic burden remains largely unknown. *Methods and Materials*. We analyzed subjects undergoing coronary calcium score evaluation by computed tomography (MDCT) and fatty liver assessment using abdominal ultrasonography. Framingham risk score (FRS) and metabolic risk score (MRS) were obtained in all subjects. A graded, semiquantitative score was established to quantify the severity of NAFLD. Multivariate logistic regression analysis was used to depict the association between NAFLD and calcium score. *Results*. Of all, 342 participants (female: 22.5%, mean age: 48.7 ± 7.0 years) met the sufficient information rendering detailed analysis. The severity of NAFLD was positively associated with MRS (*X* ^2^ = 6.12, trend *P* < 0.001) and FRS (*X* ^2^ = 5.88, trend *P* < 0.001). After multivariable adjustment for clinical variables and life styles, the existence of moderate to severe NAFLD was independently associated with abnormal calcium score (*P* < 0.05). *Conclusion*. The severity of NAFLD correlated well with metabolic abnormality and was independently predict coronary calcification beyond clinical factors. Our data suggests that NAFLD based on ultrasonogram could positively reflect the burden of coronary calcification.

## 1. Introduction

Atherosclerosis is the most common vascular pathology in patients with cardiovascular events, which leads to mortality in developed countries. There are various tools to evaluate the diseased populations for prediction, diagnosis, and risk stratification. Among asymptomatic patients, the predictive and prognostic values of calcium scores via electron beam computed tomography (EBCT) were proved in previous studies, either as an addition to the FRS or to C-reactive protein [1], or used alone [2]. Because coronary calcification is not uncommon in patients with myocardial ischemia, multidetected computed tomography (MDCT) has become a more useful modality for coronary heart disease evaluation. On the other hand, evolving evidence showed that NAFLD may actually represent metabolic syndrome in liver [3]. The existence and severity of NAFLD by liver biopsy as a gold standard also proved to be association with MRS [4, 5]. Recent studies demonstrated that NAFLD could further predict cardiovascular diseases or even involved the pathophysiologic process of atherosclerosis [4]. 

 In our study, we sought to define the correlation between the extent of NAFLD, cardiovascular risks, and MDCT-acquired calcium score. We also tried to examine the role of NAFLD beyond other clinical factors in the prediction and identification of coronary calcification. 

## 2. Materials and Methods

### 2.1. Study Subjects

We subsequently enrolled 342 non-alcoholism from outpatient clinics or subjects underwent health evaluation who had both MDCT examination for cardiovascular risk stratification as well as abdominal ultrasonography for the detection of fatty liver disease. This study was proved by the ethics committee of Mackay Memorial Hospital, Taipei, Taiwan (09MMHIS038), in accordance with the Declaration of Helsinki.

### 2.2. Data Collection and Laboratory Parameters

Anthropometric measurements including body height, body weight, waist circumference, and blood pressures at rest were taken by a registered technician who was blinded to the other test results. Body mass index (BMI) was derived from the ratio of weight to height squared. Body surface area (BSA) was calculated according to formula of DuBois. Review of medical history, physical examination, 12-lead electrocardiogram, and chest plain film were all performed by study physicians. High-sensitivity C-reactive protein (hs-CRP) levels were determined by a latex particle-enhanced immunoassay using Elecsys 2010 method (Roche, Mannheim, Germany). The other laboratory data, including a lipid profile and a renal function test, was obtained by a Hitachi 7170 Automatic Analyzer (Hitachi Corp. Hitachinaka Ibaraki, Japan). Immunoreactive insulin was measured by radioimmunoassay (PerkinElmer Automatic Gamma Counter 1470; PerkinElmer, Waltham, MA, USA). Insulin resistance was further defined as using the following homeostasis model equation: insulin resistance method (HOMA-IR) = fasting glucose (mmol/L) × fasting insulin (*μ*U/mL)/22.5. HbA1c level was obtained by high-performance liquid chromatography (Bio-rad Variant II; Bio-Rad Laboratories, Hercules, CA, USA). Body fat composition percentage was assessed by using a commercialized foot-to-foot bioelectrical impedance scale (Tanita Inc., Tokyo, Japan, models TBF 410GS).

### 2.3. Abdominal Ultrasonography for NAFLD Grading

Abdominal ultrasonography is a validated tool to diagnose fatty liver rather than liver biopsy. The presence of fatty liver disease was detected using abdominal ultrasonography done by an experienced gastroenterologist who has no reference of the participants' other data. Three levels adopted for evaluating the severity of fatty liver was based on 4 basic techniques (hepatorenal echo contrast, liver brightness, deep attenuation, and vascular blurring) as described from previous studies [6]. Right kidney echogenicity was used for the contrast of liver parenchyma echogenicity. It is normal with the same kidney cortex and liver parenchyma echogenicity. The severity of NAFLD is graded as the following: mild: minimal diffuse increase in liver brightness, but diaphragm and intrahepatic vessel contours seem normal; moderate: medium grade diffuse increase in liver brightness and mild attenuation of diaphragm and intrahepatic vessels; severe: apparent increase in echogenicity. Under the aware that fatty liver interpretation by using ultrasonic assessment may be subjective, we adopted a more strict methodology in categorizing subjects with at least moderate degree fatty liver disease as significant fatty liver disease in this study.

### 2.4. Coronary Calcium Score Measurement

Coronary calcification of coronary arteries was quantified by using a dedicated offline workstation (Aquarius 3D Workstation, TeraRecon, San Mateo, CA, USA). A coronary calcified lesion was defined as an area with a density >130 HU and covering at least 6 pixels. Scanning was performed by a 16-slice MDCT scanner (Sensation 16, Siemens Medical Solutions, Forchheim, Germany) with 16 × 0.75 mm  collimation, rotation time 420 ms,  and tube voltage of 120 kV  in one breath hold. From the raw data, the images were reconstructed with standard kernel in 3 mm thick axial, nonoverlapping slices and 25 cm  field of view. The Agatston score method was applied by multiplying each lesion (area) by a weighted CT attenuation score in the lesion.

### 2.5. Definition of Framingham Risk Score

The Framingham risk score (FRS) is designed to estimate 10-year risk of developing coronary heart disease (myocardial infarction and coronary death) in adults aged 20 and older who do not have heart disease or diabetes. This tool is based on the score (Adult Treatment Panel III, ATP-III) deriving from the information of age, gender, cholesterol dysregulation, (pre) hypertension, and smoking [7].

### 2.6. Definition of Metabolic Risk Score

Similar to ATP-III criteria, abnormal metabolic components defined by the Bureau of Health Promotion, Department of Health, Taiwan were [8]: (1) increased waist circumference of at least 90 cm  in men and of at least 80 cm  in women; (2) abnormally elevated serum triglycerides (TG) of at least 150 mg /dL; (3) lower serum high-density lipoprotein (HDL) cholesterol of less than 40 mg /dL for men and less than 50 mg /dL for women; (4) higher fasting blood glucose level greater than 110 mg /dL; (5) elevated systolic blood pressure of at least 130 mmHg  and/or diastolic blood pressure of at least 85 mmHg , or undergoing hypertension treatment. The scoring system was calculated and presented as the numbers of abnormal items meeting the criteria, with score 0 for the absence and score 5 for the presence of all 5 abnormal metabolic components. Metabolic risk score (MRS) of 3 or more met the definition of metabolic syndrome.

### 2.7. Statistics

All data was presented as mean ± SD. Continuous data between groups with and without abnormal calcium score deposition were compared by using Mann-Whitney test with abnormal distribution and by Student *t-*test if in normal distribution. Categorical variables were compared by Chi square and Fisher Exact tests as appropriate. Nonparametric rank sum test was used to test the graded change of cardiovascular risks estimated by using ATP III or Framingham system over different fatty liver status. Multivariable regression model was used based on Framingham system (FRS) and ATP III (MRS) with life styles or other variables not included in the Framingham system (FRS) or ATP III (MRS) model sequentially entered to identify the independent value in predicting fatty liver disease. Receiving operative characteristic (ROC) curves were used to test the hypothesis that whether the existence of NAFLD helps provide incremental value in the detection of abnormal coronary calcium deposition beyond traditional cardiovascular risk systems. *P*  value was set at two-tailed probability, and a *P* value less than 0.05 was considered statistically significant. All analysis was done by the software package STATA 8.2 (StataCorp, College Station, TX, USA). 

## 3. Results

### 3.1. Patients Demographics and Baseline Characteristics

Of all 342 participants, 239 subjects (mean age 47 years, 27.6% female) did not have notable calcification of all coronary territories (Group I), whereas 103 subjects (mean age 52.6 years, 10% female) were with obvious coronary calcification (Group II). Main demographic data and baseline characteristics are illustrated in Table 1. Subjects with abnormal calcium scores (Group II) were older with more male gender. In addition, they tended to have larger BMI, waist circumference, and higher blood pressure. Baseline biochemistry did not show significant differences between these two groups except a trend toward higher serum glucose level. MRS and FRS were also higher in group II. The prevalence of abnormal electrocardiographic patterns like ischemia or hypertrophy was higher in group II. Subjects in group II were also observed to have higher prevalence of hypertension, diabetes or hyperlipidemia medical history when compared with group I.

### 3.2. The Independent Predictive Value of NAFLD in Different Models

**Figure 1:**
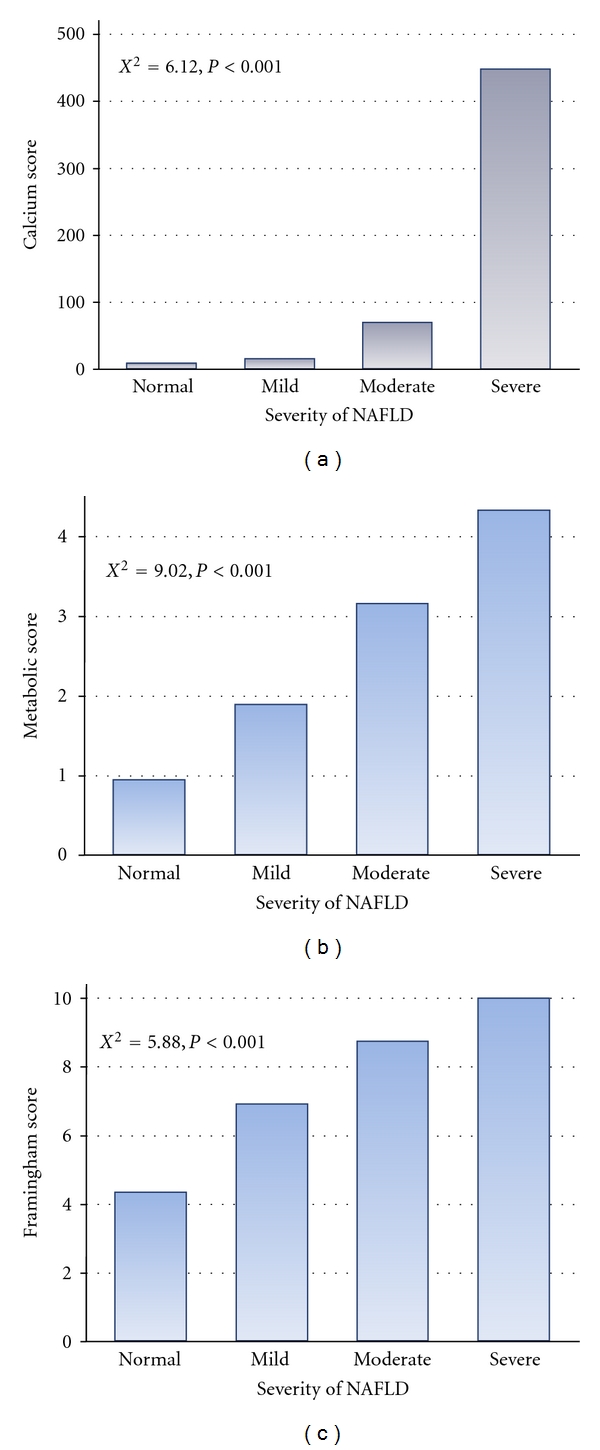
Estimated cardiovascular risk scores and coronary calcification categorized by the severity of nonalcoholic fatty liver disease. More severe fatty liver disease was associated with higher cardiovascular risk scores by either metabolic or Framingham risk scores and coronary calcium scores.

In Figure 1, we illustrated the relationship between coronary calcium score, cardiovascular risk scores, and the degree of fatty liver echogenicity. More severe fatty liver degree was observed to be associated with higher calcium scores, higher FRS and MRS (trend *P* < 0.001). We further examined the independent value of NAFLD in the prediction of abnormal coronary calcium scores by testing different clinical variables based on FRS or MRS separately into three different models (Table 2). In model 1, when other clinical variables including smoking and exercise based on the relatively insufficiency of individual scores were entered into regression model, significant fatty liver disease independently identified coronary calcium existence in either FRS group or the MRS group. In model 2, when liver function tests were together in regression model, moderate to severe fatty liver disease was still independently associated in both groups. In model 3, when body fat composition was further entered in the regression model, the presence of fatty liver disease remained statistically significantly independent in the prediction of coronary calcium existence.

### 3.3. The Incremental Value of NAFLD beyond Traditional Cardiovascular Risks in the Prediction of Coronary Calcium Scores

**Figure 2:**
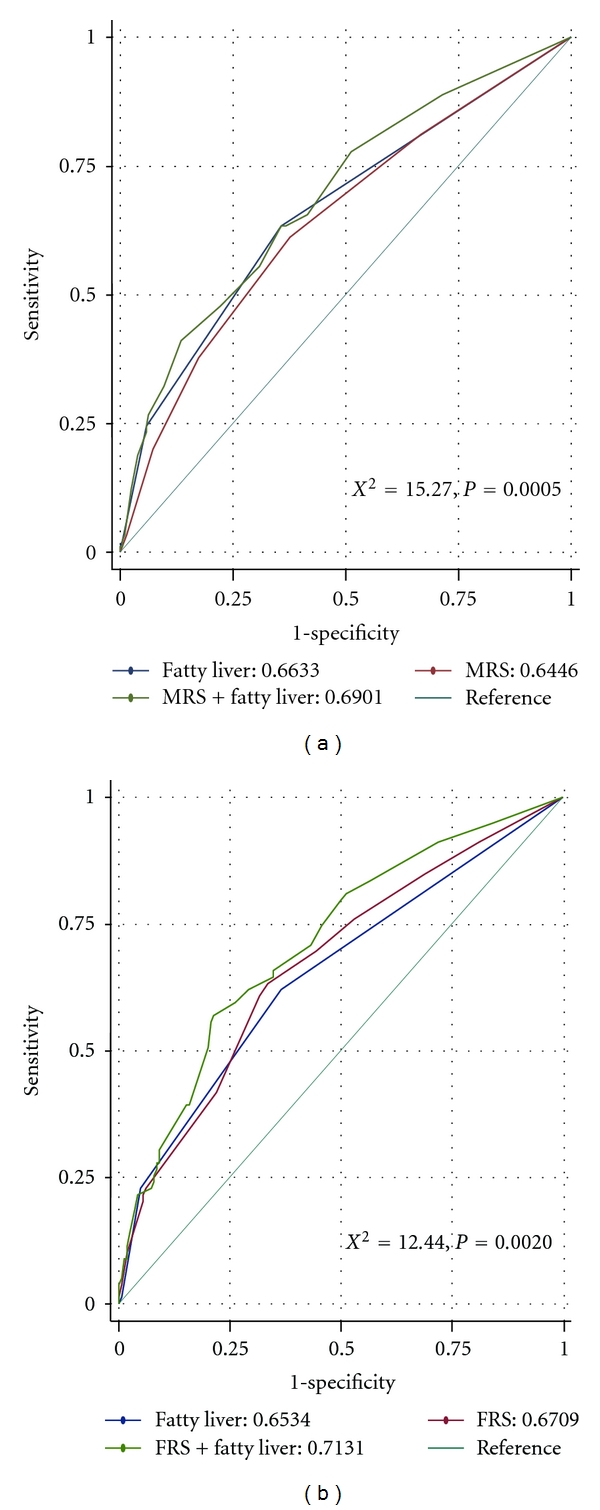
Receiver operating characteristic curves demonstrated by both metabolic or Framingham risk scores and superimposed by fatty liver disease in predicting coronary calcium deposition. When presence of fatty liver disease was added on traditional cardiovascular risk scores, there was significant increase in the area under curve.

In Figure 2, we tested the hypothesis that whether the presence of fatty liver disease could further expand the area under curve (AUC) for the discrimination of coronary artery calcification from normal subjects meaningfully. While FRS and MRS alone have an AUC of 0.67 and 0.64, the addition of NAFLD further significantly expanded the AUC to 0.71 and 0.69, respectively (*c*-statistics < 0.05).

## 4. Discussion

In this study, anthropometric data from asymptomatic people without known cardiovascular disease demonstrated a significantly positive correlation between coronary calcium score and traditional anthropometrics, blood pressure, insulin resistance, and systemic inflammation marker in terms of hs-CRP. Both estimated cardiovascular risk scores including FRS and MRS for the prediction of coronary calcification in our study by utilizing ROC analysis were similar to that in Wanamethee's report [9]. However, unique to this study, we found that more severe fatty liver disease, when assessed by abdominal ultrasonography, may serve as an independent factor even after adjustment of clinical variables and estimated cardiovascular risk scores. Furthermore, we also demonstrated that the presence of more severe degree fatty liver disease added incremental value beyond such traditional cardiovascular risks in the prediction of coronary artery calcification.

Atherosclerosis, conceptualized as a convergence of bone biology with vascular inflammatory pathobiology [10], can recently be assessed and quantified by extent of coronary artery calcification in terms of coronary calcification score when EBCT was clinically introduced and started to serve as a feasible surrogate marker [11–13]. Higher calcium scores are seen in most patients with myocardial ischemia, either symptomatic or silent [2, 14]. And the result of coronary calcium score derived from EBCT also predicts stress-related ischemia on stress nuclear images [14] and future cardiovascular events [2].

Metabolic syndrome as a medical disorder complex with a central key factor of obesity accompanied with insulin resistance has been proved as an antecedent of types of diabetes mellitus and several cardiovascular diseases [9, 15–20]. Another frequently used cardiovascular risk estimate such as Framingham score is also a widely accepted scoring system, using age, smoking, HDL, BP, and cholesterol instead of triglyceride, in predicting cardiovascular risks [1, 9]. The ATP III had suggested usage of both the metabolic risk factors plus Framingham score in determining the risk of cardiovascular events and targeting treatment goal of LDL. It is thus not surprising that NAFLD, defined as excess fat accumulation in the fat-laden hepatocytes by light microscopy which covers a broad clinical spectrum of liver diseases, be viewed as a component or consequence of metabolic syndrome [21, 22]. However, this “gold standard” is not clinically feasible in daily practice. In the recent years, liver ultrasound has emerged as the most common and simplest one of the alternative tools in NAFLD diagnosis [23].

In contrast to Caucasian, the Asian populations have shown higher prevalence of nonalcoholic steatohepatitis than alcoholic hepatitis with nearly 33% of NAFLD meet the complete diagnosis of metabolic syndrome [24]. In this regard, NAFLD has thus been deemed a hepatic representation of metabolic syndrome [3].

Recent studies have demonstrated that NAFLD patients had developed subclinical atherosclerosis when compared to nonsteatosis individuals. Further, cardiovascular disease is the second most common cause of death in NAFLD patients [4]. More importantly, subjects with known steatosis are associated with abnormal carotid intima-media [25], more vulnerable coronary plaques by MDCT [26], higher serum markers of inflammation [27], worse endothelial function, increased myocardial insulin resistance [28], decreased adiponectin concentrations, and abnormal lipoprotein metabolism [29–31].

Ethnic differences in MESA study, such as higher percentage of coronary calcification in Chinese than Hispanic and black groups, was suggestive of unknown mechanism influencing cardiovascular diseases [32]. So far, it remains inconclusive and controversial regarding the true causal relationship between NAFLD and cardiovascular diseases. Some studies had ever described that NAFLD is less likely a direct mediator of cardiovascular disease but an “epiphenomenon" [33]; however, our study reported the independent role of NAFLD in predicting coronary calcification beyond traditional cardiovascular scores. This finding may have an impact on cardiovascular risk stratification or even disease process evaluation. More severe form of NAFLD could thus be viewed as an independent clinical marker for higher cardiovascular risks that may benefit from a more aggressive approach. It also deserves more efforts to work out the influence of liver fat intervention on cardiovascular diseases compared to prior reports [34, 35].

## 5. Conclusion

The severity of NAFLD not only links to metabolic derangement and traditional cardiovascular risks but also independently identifies the burden of coronary atherosclerosis in terms of coronary calcification. Simple NAFLD grading by liver ultrasonography may serve as a clinically useful tool beyond conventional risk factors in cardiovascular risk stratification.

## Figures and Tables

**Table 1:** Baseline demographic data and clinical information in our study.

	Calcium score zero (*N* = 239)	Calcium score abnormal (*N* = 103)	*P* value
Age, years	47.0 (6.4)	52.6 (6.6)	<0.001
Gender, female %	66 (27.6)	11 (10.6)	
Height, cm	165.9 (7.5)	167.7 (7.5)	0.09
Weight, kg	65.9 (11.3)	68.9 (10.1)	0.008
BMI, Kg/m^2^	23.8 (3.1)	24.4 (2.7)	0.013
Waist, cm	81.4 (9.1)	85.0 (7.9)	<0.001
Waist to hip ratio	0.88 (0.07)	0.91 (0.06)	<0.001
SBP, mmHg	116.7 (14.9)	125.1 (15.1)	0.003
DBP, mmHg	74.5 (10.2)	77.9 (9.7)	0.002
Biochemistry			
Sugar (AC), mg/dL	93.3 (21.2)	102.0 (20.9)	0.086
Sugar (PC), mg/dL	105.9 (31.6)	118.5 (44.4)	0.082
HbA1c, %	5.8 (0.6)	6.0 (0.6)	0.32
Insulin, U/mL	6.25 (4.24)	6.91 (3.75)	0.046
HOMA-IR	1.55 (1.25)	1.76 (1.06)	0.016
Cholesterol, mg/dL	191.1 (32.9)	196.1 (35.1)	0.23
LDL, mg/dL	128.0 (31.3)	125.8 (31.7)	0.56
HDL, mg/dL	53.0 (13.7)	49.1 (12.8)	0.02
TG, mg/dL	141.9 (103)	145.8 (85.2)	0.74
BUN, mg/dL	11.8 (3.2)	12.6 (3.7)	0.32
Creatinine, mg/dL	0.91 (0.18)	0.97 (0.18)	0.39
Uric Acid, mg/dL	5.6 (1.4)	6.1 (1.5)	0.003
Homocystine, mg/dL	7.3 (2.1)	8.4 (2.3)	0.03
Hs-CRP, mg/dL	0.2 (0.35)	0.32 (0.7)	0.043
ECG pattern			
ECG (LVH or myocardial ischemia)	41 (7.5)	28 (13.2)	0.016
ECG (old infarct)	5 (1)	9 (4.2)	0.002
Medical history			
Smoking	84 (24.9)	42 (29.4)	0.311
HTN history	41 (12)	35 (23.7)	0.001
DM history	11 (3.2)	14 (9.5)	0.004
Hyperlipidemia	17 (5.6)	13 (9.6)	0.039
CVD	12 (3.9)	12 (8.9)	0.033
Family Hx			
Family Hx HTN	147 (43)	62 (41.9)	0.823
Family Hx DM	88 (25.8)	42 (28.4)	0.554
Family Hx Stroke	49 (14.3)	20 (13.5)	0.812
Family Hx CVD	57 (16.7)	30 (20.3)	0.345

BMI: body mass index; SBP: systolic blood pressure; DBP: diastolic blood pressure; sugar (AC): fasting glucose; sugar (PC): postprandial glucose; HOMA-IR: insulin resistance; ECG: electrocardiography; LVH: left ventricular hypertrophy; HTN: hypertension; DM: diabetes; NAFLD: nonalcoholic fatty liver disease; CVD: cardiovascular disease: Hx: history.

**Table 2:** Multivariate logistic regression models in predicting coronary calcium deposition.

Variables	OR	SE	*Z*	*P*	CI 95%	
*Model 1*						
FRS	1.16	0.05	3.09	0.002	1.054599	1.268832
BMI	0.93	0.06	−1.23	0.218	.8193498	1.04653
Sugar (AC)	0.99	0.01	−0.5	0.618	.9744172	1.015514
Drinking	0.99	0.39	−0.02	0.981	.4557979	2.153525
Exercise	0.76	0.63	−0.33	0.742	.1488091	3.882643
Fatty liver (moderate to severe)	4.47	2.66	2.52	0.012	1.393377	14.33824
DM history	1.92	2.15	0.58	0.563	.2114827	17.35303

MRS	1.1	0.15	0.67	0.506	.836501	1.436341
Age	1.16	0.03	5.16	<0.001	1.097237	1.229447
Sex	4.84	2.62	2.92	0.004	1.677794	13.97886
Drinking	0.7	0.28	−0.88	0.381	.3176925	1.549465
Smoking	0.8	0.36	−0.49	0.627	.3329831	1.94089
Exercise	0.32	0.3	−1.21	0.225	.0488721	2.032724
Fatty liver (moderate to severe)	3.83	2.13	2.42	0.016	1.288897	11.39297

*Model 2*						
FRS	1.15	0.06	2.87	0.004	1.045555	1.26785
BMI	0.92	0.06	−1.18	0.24	.811891	1.053551
Sugar (AC)	0.99	0.01	−0.71	0.478	.9715331	1.013632
Drinking	0.77	0.33	−0.61	0.544	.3299596	1.794328
Exercise	0.78	0.67	−0.29	0.772	.145282	4.191667
Fatty liver (moderate to severe)	7.36	5.35	2.75	0.006	1.769904	30.56831
DM history	1.71	2	0.46	0.649	.1713221	16.99859
Viral hepatitis carrier	1.14	0.65	0.23	0.82	.3714342	3.491325
GPT	0.99	0.01	−0.92	0.359	.9695872	1.011263
rGT	1.02	0.01	1.1	0.272	.9879836	1.043843

MRS	1.06	0.16	0.37	0.71	.7835745	1.43072
Age	1.16	0.03	4.93	<0.001	1.092158	1.226692
Sex	5.19	2.9	2.95	0.003	1.737443	15.52867
Drinking	0.58	0.25	−1.26	0.207	.2466988	1.353505
Smoking	0.8	0.38	−0.47	0.641	3155051	2.035192
Exercise	0.29	0.27	−1.31	0.19	.0437895	1.8624
Fatty liver (moderate to severe)	7.43	4.95	3.01	0.003	2.014803	27.42179
Viral hepatitis carrier	1.34	0.77	0.51	0.613	.4322222	4.144012
GPT	0.98	0.01	−1.41	0.159	.9619023	1.006359
rGT	1.01	0.01	1.01	0.312	.9861562	1.044676

*Model 3*						
FRS	1.14	0.05	2.78	0.005	1.040021	1.253657
BMI	1	0.08	0.1	0.921	.8635472	1.176196
Sugar (AC)	0.99	0.01	−0.67	0.505	.9722974	1.013941
Drinking	0.73	0.32	−0.74	0.461	.3102782	1.70035
Exercise	0.4	0.39	−0.94	0.347	.0574678	2.726738
Fatty liver (moderate to severe)	6.77	4.95	2.62	0.009	1.61448	28.35676
DM history	1.46	1.68	0.33	0.743	.1532743	13.87366
Viral hepatitis carrier	1.31	0.77	0.47	0.641	.418516	4.118456
GPT	0.99	0.01	−0.87	0.385	.9708703	1.011461
rGT	1.02	0.01	1.03	0.302	.9870173	1.043093
Body fat	0.92	0.04	−1.94	0.053	.8456979	1.000979

MRS	1.12	0.18	0.72	0.473	.8203864	1.532548
Age	1.16	0.03	4.97	<0.001	1.092734	1.226381
Sex	3.1	2	1.76	0.078	.8790155	10.95577
Drinking	0.58	0.25	−1.25	0.211	.2463022	1.363246
Smoking	0.83	0.4	−0.38	0.702	.3256642	2.129526
Exercise	0.18	0.18	−1.75	0.08	.0260914	1.227528
Fatty liver (moderate to severe)	8.58	6	3.07	0.002	2.176022	33.82722
Viral hepatitis carrier	1.44	0.84	0.63	0.528	.4627544	4.488757
GPT	0.99	0.01	−1.17	0.243	.9656288	1.008901
rGT	1.02	0.02	1.04	0.298	.9864816	1.045414
Body fat	0.94	0.04	−1.57	0.117	.8679529	1.015836

FRS: Framingham risk score; MRS: metabolic risk score; *γ*-GT: *γ*-glutamyl transpeptidase; GPT: alanine aminotransferase. Other abbreviations were listed in Table 1.
